# The London Municipalities

**Published:** 1900-10-27

**Authors:** 


### The Hospital

A Journal of

The Medical Sciences and Hospital Administration.

Vol. XXIX.—No. 735.] EDITED BY SIR HENRY BURDETT, K.C.B., AND SOLOMON C. SMITH, M.D., M.R.C.P. [October 27, 1900.

### The London Municipalities.

The question whether the brand-new municipalities about to be provided for the Metropolis will be anything more than Bumbledon writ large is one of very considerable importance to the health, the pockets, and the general welfare of some five millions of people; and the manner in which it is determined can hardly fail to exercise an appreciable influence over the fortunes of a still larger number. The new boroughs may either retain "parochialism" at its existing level, or may lift it to one better adapted to the requirements of the twentieth century. Whichever they do, they will furnish an example, either of "how to do it" or of "how *not* to do it," which is likely to be extensively followed in the country at large, and to produce effects of a decided and far-reaching character. Many provincial towns have of late years afforded admirable precedents, by persuading territorial magnates in their vicinity to accept the office of mayor, and, in some cases, even to discharge its duties with scrupulous attention and fidelity; with the obvious effect of increasing the dignity which had previously attached to it, and of rendering it, more than formerly, an object of ambition to men of public spirit. So much depends, in every case, upon a good beginning, that it is greatly to be hoped that the new London boroughs may be able to follow and to extend this precedent, and to find within their boundaries men of high rank, as well as of distinguised character and position, who will consent to dignify municipal office by accepting it, and who will accept it with a determination to acquire mastery of its details, and to watch closely over the best interests of their constituencies. It is perhaps unfortunate that the municipal elections have been so immediately preceded by the election of the new House of Commons; a circumstance which cannot fail greatly to facilitate the application, to the former, of organisations created and maintained chiefly with reference to the latter. The aims in the two cases should be widely different, and the methods of securing them should reflect the difference. Nineteen members of the House of Commons out of twenty can hardly be regarded in any more serious light, to use Mr. Chamberlain's felicitous phrase, than as "units in a voting machine," and the chief desire of the purveyors of candidates is to find units who may be relied upon to "vote straight," if they are elected, and to leave the task of thinking to their official leaders, or misleaders. In a municipality the case varies widely from this, the smaller number of members permitting greater elbow-room to individuals, and the division of labour rendered practicable by committees not only increasing this elbow-room, but also affording scope for the employment of many kinds of special knowledge in different departments, each of which becomes contributory to the advantage of the whole. The legislation of Parliament is largely an assertion of principles, which must be defined by leaders and can only be accepted by followers. The work of a municipality is chiefly the application of these principles to particular cases, or the adaptation of them to circumstances which may vary in different localities. In the performance of this work, the talent and capacity of every individual concerned may have ample scope; and the aggregate amount of talent and capacity will be likely to exert a great and abiding influence upon the results produced. The introduction into such a body of mere political units, or the bestowal of its seats as consolation prizes for disappointed local politicians, could scarcely fail to exercise a disastrous influence upon its efficiency, and to deprive the public of the benefits which it has been designed in order to secure. There is only too much reason to fear that arrangements for such an introduction or bestowal have been far advanced in many constituencies, or that urgent endeavours will be made to render the municipality a haven of refuge for politicians who have failed to obtain entrance to the London County Council. Against such a calamity it behoves everyone to strive, if for no other reason, because the public health of the metropolis is certain to be materially influenced by the choice of the electors. There is scarcely one among the prominent duties of a municipality, such as lighting, paving, cleansing, or whatever it may be, which does not, at least indirectly, and often directly and seriously, affect the sanitary state within its area, and, therefore, for good or for evil, not only the comfort, but also the health, of the subject population. A corrupt or even a merely ignorant and obstinate borough councillor may be a small thing in itself, but, either in the way of furtherance or of impediment, he may assist in bringing about very undesirable results. The difficulty of making a wise selection, or of inducing the most desirable men to offer themselves as candidates, is greatly due to the combined effects of extent of surface and density of population; circumstances which render it almost impossible for any resident, who does not fill a conspicuous position in the public eye, to be sufficiently well known to his neighbours to enable them really to estimate his suitability. Under the rule of vestrydom, which is passing away, every ratepayer received a blue list of candidates from one political organisation, and a red list from another, with a request to vote for it *en bloc*. As a rule, he would never have heard of either the red or the blue people until their names were thus brought under his notice, and he would have no real knowledge of either their powers or their sentiments. It is greatly to be hoped that some better method of procedure may now come to prevail in the election of the new municipal bodies.

